# Acceptability and Preliminary Results of Technology-Assisted Balance Training in Parkinson’s Disease

**DOI:** 10.3390/ijerph19052655

**Published:** 2022-02-24

**Authors:** Elvira Maranesi, Valentina Di Donna, Giuseppe Pelliccioni, Valentina Cameriere, Elisa Casoni, Renato Baldoni, Marco Benadduci, Nadia Rinaldi, Lorenzo Fantechi, Cinzia Giammarchi, Riccardo Luzi, Paolo Pelliccioni, Mirko Di Rosa, Pietro Scendoni, Giovanni Renato Riccardi, Roberta Bevilacqua

**Affiliations:** 1 Scientific Direction, IRCCS INRCA, 60124 Ancona, Italy; m.benadduci@inrca.it (M.B.); c.giammarchi@inrca.it (C.G.); r.bevilacqua@inrca.it (R.B.); 2 Clinical Unit of Physical Rehabilitation, IRCCS INRCA, 63900 Fermo, Italy; v.didonna@inrca.it (V.D.D.); n.rinaldi@inrca.it (N.R.); p.scendoni@inrca.it (P.S.); 3 Neurology Unit, IRCCS INRCA, 60127 Ancona, Italy; g.pelliccioni@inrca.it (G.P.); v.cameriere@inrca.it (V.C.); 4 Clinical Unit of Physical Rehabilitation, IRCCS INRCA, 60127 Ancona, Italy; e.casoni@inrca.it (E.C.); r.baldoni@inrca.it (R.B.); g.riccardi@inrca.it (G.R.R.); 5 Clinical Unit of Nuclear Medicine, IRCCS INRCA, 60127 Ancona, Italy; l.fantechi@inrca.it; 6 Medical Direction, IRCCS INRCA, 60127 Ancona, Italy; r.luzi@inrca.it; 7 Eye Clinic, Polytechnic University of Marche, 60127 Ancona, Italy; paopel@hotmail.it; 8 Unit of Geriatric Pharmacoepidemiology, IRCCS INRCA, 60124 Ancona, Italy; m.dirosa@inrca.it

**Keywords:** older people, Parkinson’s Disease, balance, gait speed, technology-based intervention

## Abstract

(1) Background: Parkinson’s Disease (PD) is one of the most common causes of disability among older individuals. The advanced stages of PD are usually characterized by postural instability and, as a consequence, falls. Those are among the main factors that determine the quality of life, as well as the morbidity and mortality of a person with PD. In the field of PD rehabilitation, robotics is also rapidly gaining ground. As a primary aim, we evaluate the acceptability of the technology integrated intervention, using the Psychosocial Impact of Assistive Devices Scale (PIADS), in order to analyze the attitude of the participants towards the Tymo^®^ system. As a secondary outcome, we assess the result of the rehabilitation treatment integrated with the Tymo^®^ system on several patient’s features. (2) Methods: We studied a population of 16 patients with Parkinson’s Disease. Each recruited subject completed 10 treatment sessions, organized as two training sessions per week, for 5 weeks. The intervention included 30 min of traditional therapy and 20 min of technological treatment with a robotic system. PIADS is composed of three subscales (Competence subscale, Adaptability subscale, Self-esteem subscale) ranging from −3 to +3, reflecting, respectively, a negative or positive feeling towards the device. (3) Results: The Competence subscale, measuring feelings of competence and usefulness, obtained a score of 1.24 (SD = 0.78). The score of Adaptability subscale, indicating a willingness to try out new things and to take risks, was 1.83 (SD = 0.65). Finally, the Self-esteem subscale, indicating feelings of emotional health and happiness, reached a score of 1.31 (SD = 0.72). Moreover, statistical analysis reveals a significant effect on balance performance after intervention. (4) Conclusions: This feasibility study represents a starting point in the use of technology in the rehabilitation pathway of patients affected by Parkinson’s Disease. In fact, our results suggest that a standard therapy combined with an innovative treatment using Tymo^®^ may be accepted by PD patients, which may benefit especially from preserving balance.

## 1. Introduction

Parkinson’s Disease (PD) is one of the most common causes of disability among older persons [1]. It is a chronic-neuro-degenerative disease that progresses with time, characterized by motor disorders, such as bradykinesia (poor and slow movement), tremor at rest, rigidity, posture in flexion and shuffling gait; in the late stages of the disease, there is the presence of postural instability leading to a deficit of balance and as a consequence, a high risk of falling [2]. In fact, postural instability is one of the cardinal signs in PD and can be present even at diagnosis, but becomes more prevalent and worsens with disease progression [3]. Moreover, in the early stages of PD, balance assessment was shown to quite reliably differentiate it from early atypical parkinsonisms such as progressive supranuclear palsy (PSP), improving our physiological understanding of balance disorders in parkinsonian disorders [4]. Balance is an essential prerequisite that needs a multifaceted network of sensory information integrations, aimed at the realization of a subjective perception of body position, relative to the surrounding environment, in order to generate an appropriate motor response that controls body movements. 

Aging, neuroinflammation and microglial activation, in particular in neurodegenerative diseases, leads to a progressive loss of function of the neuromotor system, and this phenomenon can contribute to worsening the balance deficit [1,5,6]. Postural instability is considered a motor symptom that is potentially related to dysfunctional mesencephalic neurotransmission, such as degeneration of cholinergic neurons in the pedunculopontine nucleus [7,8,9]. The disturbance of balance occurs later in the course of the disease and is a symptom that involves the “axis of the body”; it is due to a reduction of the straightening reflexes, so that the subject is not able to spontaneously correct any imbalance. It can be evidenced while walking, also when the person changes direction. Postural instability enhances the risk of falling, which in turn, is associated with subsequent injuries and further disability [10,11,12]. Balance disorders do not respond to the dopaminergic therapy used in PD [13], and levodopa may increase falls in these patients [14]. There are conflicting reports of levodopa effectiveness on balance and gait, with some authors stating that gait over balance improvement might explain why, in some patients, levodopa may increase falls [14].

Novel interventions are needed to improve the adaptations to postural perturbations. Therefore, physiokinesiotherapy becomes a meaningful intervention for the management of motor disturbances. In rehabilitation settings, the effects of bradykinesia, stiffness, impaired proprioception, postural instability, and freezing of gait in parkinsonian patients have long been studied and are well known [5,10,15]. Postural instability and, as a consequence, falls, are among the principal factors that determine the quality of life, as well as the morbidity and mortality of a person with PD [5,12,16]. In fact, the Koninklijk Nederlands Geleidehonden Fonds (KNGF) guidelines for physical therapy in PD recommend that, from the onset of the disease, patients undergo a falls prevention course [16].

Rehabilitation approaches, aimed at improving static/dynamic balance, recovery of ambulation, and prevention of falls, are the usual treatment for those pathological conditions [5,10,12,16,17]. Originally, such rehabilitation approaches were based on empirical experience, but increasing scientific evidence suggests that “exercise-dependent” neuronal plasticity is the main mechanism supporting the efficacy of physiotherapy treatment [18,19,20,21,22,23]. Exercise increases synaptic connections and influences neurotransmission, enhancing functional PD circuits. In fact, exercise-induced cortical plasticity is reflected by means of stimulus-dependent alterations in brain areas shown by magnetic resonance imaging in healthy volunteers, and there is increasing data suggesting that the human brain is capable of undergoing learning-related structural plasticity even in a pathophysiological disease state such as in PD [18,19,20,24]. Recent meta-analyses show that rehabilitation brings important clinical benefits in walking speed and balance [19,20,22,23]; recent evidence suggests that balance training might improve this motor disability [25]. In addition, some authors highlight the importance of initiating the exercises-based intervention in mild stages of the disease, suggesting the positive role of external visual and auditory cueing systems to recover from balance difficulties [26].

In the field of PD rehabilitation, technology is also rapidly gaining ground [20,21,22,27,28]. Different contributions [19,20,21] show that technology-based balance training induces performance improvements, which also correlate with clear neurobiological changes in the cerebral cortex in functional magnetic resonance (fMR) [19,20]. These innovative strategies in rehabilitation request more research in the evaluation of the short- and long-term effects in patients over 65 years of age with PD, and first of all, the assessment of their feasibility and sustainability for the health providers. Our purpose is to investigate and clarify whether an innovative technology-based intervention focused on static balance training (Tymo^®^ stabilometric platform) can be integrated in a traditional physiotherapy intervention, aimed at improving the kinematic/kinetic parameters of gait and static-dynamic balance. As a primary aim we evaluate the acceptability of the technology integrated intervention, in order to analyze the attitude of the participants towards the Tymo^®^ system. As a secondary outcome, we assess the result of the rehabilitation treatment integrated with the Tymo^®^ system on the gait and balance of older PD patients, the fear of falling, the level of autonomy in daily living activities and the physical and psychological state of the patients. 

## 2. Materials and Methods

The results described for this feasibility study represent the preliminary data collected for the clinical trial “Innovative Models in the Rehabilitation of the Elderly With Parkinson’s Disease Through Technological Innovation”, registered on ClinicalTrials.gov with trial registration number NCT04087031 (12 September 2019). The entire protocol, including the description of scales, the platform functioning, the training program and procedures is described in details elsewhere [29]. For this reason, the following sections are briefly summarized here.

### 2.1. Subjects

The study population consisted of patients with PD, recruited in the outpatient Departments at the Clinical Unit of Neurology and Physical Rehabilitation, IRCCS INRCA, in the Ancona and Fermo hospitals. Study participants met the following inclusion criteria: patients 65 years of age or older with early-stage Parkinson’s Disease (Hoehn and Yahr scale [30] of 1–3 stage), with the ability to give consent. In addition, patients must have a Functional Ambulation Category (FAC) ≥ 2 [31], a Rankin scale score ≤ 3 [32]; a negative Geriatric Depression Scale (GDS) 5-items evaluation [33] and a stability of drug treatment for at least 1 month.

The study was approved by the Ethic Committee of the IRCCS INRCA Hospital (process No.19017/2019). All subjects gave their informed consent prior to testing. 

### 2.2. Clinical Assessment Description

A standard assessment was performed in all patients, including: clinical history, measurement of cognition with the Mini Mental State Examination (MMSE) [34] and with the Clinical dementia rating scale (CDR) [35], evaluation of the acceptance of the technology with the Psychosocial Impact of Assistive Devices Scale (PIADS) [36], measurement of functional state with the Barthel Index (BI) [37], gait and balance performance on Tinetti’s Performance Oriented Mobility Assessment (POMA) [38], evaluation of quality of life with SF-12 health survey (SF-12) [39] and fear of falling with Falls Efficacy Scale-International (FES-I) [40]. In particular, the PIADS evaluated the effects of the prototype and training on functional independence, well-being, and quality of life. The test is composed of three subscales ranging from −3 to +3, reflecting, respectively, a negative or positive feeling towards the device (Table 1): the Competence subscale, measuring feelings of competence and usefulness, the Adaptability subscale, indicating a willingness to try out new things and to take risks, and the Self-esteem subscale, indicating feelings of emotional health and happiness. Moreover, gait analysis was performed on the patients at the Gait Analysis Laboratory in the Department of Physical Rehabilitation at a branch of IRCSS INRCA Ancona, using a G-Sensor in order to assess the gait speed. This evaluation was performed over a distance of at least 7 linear meters. Both clinical scales and gait analysis evaluation were performed during the recruitment and at the end of the treatment. The patients’ assessment was performed in two different sessions in an individual way: one was dedicated to the administration of clinical scales; another was dedicated to the execution of gait analysis. The duration of each session varied according to the subject involved but never exceeded one hour.

### 2.3. Training Program

After recruitment and administration of the clinical scales described above, each selected subject completed 10 treatment sessions, divided into two training sessions per week, for 5 weeks. The technological intervention consisted of 30 min of traditional therapy and 20 min of treatment with a robotic system. Participants had to complete at least 80% of the sessions, with the possibility of recovering a maximum of two sessions. Traditional rehabilitation treatments consisted of breathing, relaxation and postural harmonization exercises, exercises of active mobility and stretching, eye-hand coordination exercises and couple exercises, and exercises for walking and verticalization. This was performed by all patients included in the study. The intervention was carried out by a physiotherapist. Each patient performed the rehabilitation session individually in the presence of the physiotherapist who followed the movements and monitored the progress by choosing the games and levels to be performed in each session.

#### Instrumentation

The robotic treatment consisted of using the Tymo^®^ system. The Tymo^®^ system is a wireless platform used to train the person to improve their balance and postural control. The Tymo^®^ system is connected to a screen. It provides virtual reality games, which can be adapted to each patient, according to their functional capacity. For the virtual games’ execution, the patient’s body becomes the joystick that, moving in space, reaches the different objectives of the game. In this way, in addition to the motor field, the cognitive one is also involved. The physiotherapist can choose to work in one dimension (antero-posterior or medio-lateral dimension) or in two dimensions (combining the two movements) and select different games, accordingly. For this study, the platform was used in static mode.

### 2.4. Statistical Analysis

Descriptive data were presented as mean ± standard deviation (SD) for continuous variables or numbers (percentage) for categorical ones. Normality in distribution for continuous variables was assessed via a Shapiro-Wilk test. Gender differences were tested with Pearson’s chi-squared test for categorical variables and Student’s t test for continuous variables or Wilcoxon rank-sum test according to their distribution. Before/after comparison was assessed with matched-pairs Student’s *t* test or Wilcoxon matched-pairs signed-ranks test. Data were analyzed using STATA version 15.1 Statistical Software Package for Windows (Stata Corp, College Station, TX, USA).

## 3. Results

Overall, 16 patients with PD were enrolled in the study. Patients were aged 73.6 ± 8.1 years old, and consisted of 7 men and 9 women. The mean Hoehn and Yahr scale was 1.7, indicative of a unilateral and axial involvement (Table 2). 

Gender differences are statistically significantly different only in the Rankin scale values (*p* = 0.031). Specifically, men have a mean value of 1.7, indicating mild disability, while women have a mean value of 1 indicating no significant disability. Both conditions are acceptable for the study because they do not involve severe disabilities and meet the intended inclusion criteria.

Table 3 gives a summary of pre- and post-intervention scores on the functional state scales with the Barthel Index (BI), gait and balance performance on Tinetti’s Performance Oriented Mobility Assessment (POMA Gait and POMA Balance), evaluation of quality of life with SF-12 health survey (SF-12), and fear of falling (FES-I). 

Appendix A, Appendix B, and Appendix C show the value of each variable reported in the study for individual patients. In particular, Appendix A displays the values for each subject of the variables reported in Table 1; Appendix B reports the pre-intervention values, and Appendix C reports the post-intervention values for each patient of the variables reported in Table 2.

Statistical analysis reveals a significant effect on balance performance after intervention. Although an improvement in some other variables can be observed, this increase does not turn out to be significant. Moreover, the variables related to walking ability remain almost unchanged.

The subscales of PIADS obtain the following scores: the Competence subscale showed a score of 1.24 (SD = 0.78); the score of Adaptability subscale was 1.83 (SD = 0.65), and the Self-esteem subscale reached a score of 1.31 (SD = 0.72).

## 4. Discussion

The purpose of this study is to investigate and clarify whether an innovative technology-based intervention focused on static balance can be easily accepted by older patients with PD, and can show preliminary positive results in relation with gait ability, static-dynamic balance and the general status of the patients, fear of falling, and quality of life.

The acceptance of any technological solution is a crucial issue for outlining the adequacy of the systems to the patients’ needs and understanding the benefits for the health care services, especially in complex rehabilitative interventions. 

In the present feasibility study, the acceptance of the system is measured with the PIADS that takes into consideration different domains that may have an impact on the use. In the case of our study, very balanced scores were obtained for each of the subscales, in particular, a positive result was collected for the Adaptability. Within the PIADS constructs, Adaptability turns out to be specifically applicable to older users of assistive technologies, as they want a system capable of supporting the changing needs due to ageing and PD. The Adaptability subscale, in fact, is composed of six items that reflect inclination or motivation to participate socially and to adapt to activities of daily living. The other two subscales also emphasize a positive impact of technology on the patient. In fact, both the subscale on the patient’s perception of improved competence and the subscale on self-esteem have a value above 1, highlighting a positive attitude of the subject toward the system. 

Some evidence suggest that quantity and intensity of physical activity may be reduced in early Parkinson’s disease, underlined by the fact that the decline in the physical activity cannot be ascribed simply to age in the early stages of PD [41,42]. For this reason, it is necessary to engage the PD patients in rehabilitative training from the beginning of the disease and stimulating them in remaining active [43]. Numerous studies support the idea that technology-based interventions can stimulate the patients and support the long-term engagement in treatments for chronic diseases, such as PD [44]. In this way, acceptance of technology is of paramount relevance, as it may assure the use of the systems in different settings, the flexibility of the training on the basis of the patients’ needs and the engagement during their use [45]. Despite the limited sample size and the absence of a control group to support the findings, our results show an improvement of balance, after the integrated intervention. A possible explanation for this, to be necessarily re-evaluated in a more extensive randomized trial, can be determined by the engagement of the PD patients, due to technology. In fact, this type of training has the advantage of involving patients by increasing adherence to therapy in the long term [46,47]. Moreover, our method, using a combination of both cognitive and physical training, which requires the user to perform physical movements while conducting cognitive exercises, seems to be effective in the PD rehabilitation treatment [48,49]. In fact, the patient has to simultaneously perform a visual exploration task, activating visual and dual tasking cognitive control [25] to maintain balance during exercises, relying on both feedback and feedforward control. 

Some studies [19,20,21] show that technological balance training produces performance improvements that are also correlated with evident neurobiological changes in the cerebral cortex, and it is suggested that PD patients may rely on enhanced sensory information processing at least in an early phase of a whole-body dynamic balancing task learning, which in turn translates into increased structural plasticity in related brain areas [19].

Our study applies this principle with 10 treatment sessions, divided into two training sessions per week, for 5 weeks, in order to obtain benefit evidence on the functional state scales, in particular in the balance setting. We acknowledge that this study has a number of limitations that should be considered in light of the results. Firstly, the relatively small sample size may have affected our capacity to detect some relationships between cognitive function, balance, and gait, as well as the absence of a control group. In addition, the absence of a control group makes it difficult to understand the contribution that each component, traditional or technological, of the rehabilitation intervention has on improving patients’ balance. However, this feasibility study represents a first insight on the preliminary data of a more extensive randomized controlled trial. Secondly, the lack of a long-term follow-up assessment after training must be taken into consideration; evaluating the long-term impact of this type of training will be addressed in future studies. In fact, it will be interesting to evaluate whether the increase in balance of our PD patients in response to motor rehabilitation program with a device will have a long-term effect on postural instability, a symptom that is typically present only in later stages of the disease. Another important issue to be clarified will be the role of attentive and executive impairment in PD patients in postural instability [50] and the benefit of Tymo^®^, using a combination of both cognitive and physical training. Finally, the lack of significance in the other scores may be due to a ceiling effect, given that many of the subjects reached the upper limit that was set for the scale.

## 5. Conclusions

This feasibility study represents a starting point in the use of technology in the rehabilitation of the patient with Parkinson’s disease. In fact, our results suggest that a standard therapy, combined with an innovative treatment using Tymo^®^, may be accepted by PD patients that might benefit especially for preserving balance. In particular, the technological intervention was perceived as a mean to improve competence and self-efficacy of the participants as well as adaptive to their changing needs. However, the small sample size may have reduced the precision of estimates. Thus, our findings should be replicated in larger populations and compared with a control group with the same baseline characteristics, subjected to traditional treatment only. 

## Figures and Tables

**Table 1:** PIADS constructs and items.

Scale	Definition	Items
Competence	The competence subscale is composed of 12 items related to perceived functional capability, independence, and performance	Competence
		Adequacy
		Efficacy
		Productivity
		Capability
		Usefulness
		Expertise
		Performance
		Skillfulness
		Independence
		Quality of life
		Confusion
Adaptability	The adaptability subscale is composed of six items that reflect inclination or motivation to participate socially and take risks	Willingness to take chances
		Ability to participate
		Eagerness to try new things
		Ability to adapt to activities of daily living
		Ability to take advantage of opportunities
Self-esteem	The self-esteem subscale is composed of eight items reflecting self-confidence, self-esteem, and emotional wellbeing	Self-esteem
		Security
		Sense of power
		Embarrassment
		Happiness
		Sense of control
		Frustration
		Self-confidence

**Table 2:** Baseline demographic and clinical profile.

	Total *n* = 16	Male *n* = 7	Female *n* = 9	*p*
Age, mean ± SD	72.8 ± 6.3	73.8 ± 6.6	72.0 ± 6.3	0.574
Marital status, *n* (%)				0.182
Married	14 (87.5%)	7 (100%)	7 (77.8%)	
Widowed	2 (12.5%)	0 (0%)	2 (22.2%)	
Educational level, *n* (%)				0.314
Primary education	8 (50%)	2 (28.6%)	6 (66.7%)	
Secondary education	5 (31.25%)	3 (42.8%)	2 (22.2%)	
University or more	3 (18.75)	2 (28.6%)	1 (11.1%)	
Hoehn and Yahr score, mean ± SD	1.7 ± 0.7	1.7 ± 0.95	1.7 ± 0.75	0.9
Rankin scale score, mean ± SD	1.3 ± 0.7	1.7 ± 0.9	0.9 ± 0.3	0.031 ^
GDS, mean ± SD	2.4 ± 1.5	3.1 ± 1.1	1.9 ± 1.6	0.099
FAC, mean ± SD	4.6 ± 0.5	4.6 ± 0.5	4.7 ± 0.5	0.719
MMSE, mean ± SD	27.5 ± 1.9	27.7 ± 2.6	27.4 ± 1.4	0.796

SD = Standard deviation; GDS = Geriatric Depression Scale; FAC = Functional Ambulation Category; MMSE = Mini Mental State Examination; *p*-values from Pearson’s chi-squared test for categorical variables and Student’s *t* test for continuous variables; ^ *p*-values from Wilcoxon rank-sum test.

**Table 3:** Mean pre- and post-intervention scores and standard error of the mean on the BI, POMA gait and Balance, SF-12, FES-I.

	Pre	Post	p
BI	90.3 ± 11.3	93.1 ± 15.3	0.058
POMA Gait	11.0 ± 1.6	11.5 ± 1.03	0.118
POMA Balance	13.5 ± 2.3	14.5 ± 1.9	0.006 ^
SF-12	31.4 ± 2.6	30.5 ± 2.5	0.078
FES-I	11.5 ± 4.4	12.5 ± 6.1	0.640
Gait Speed [m/s]	1.9 ± 0.6	1.9 ± 0.8	0.717

BI = Barthel Index; POMA Gait = Tinetti’s Performance Oriented Mobility Assessment-Gait part; POMA Balance = Tinetti’s Performance Oriented Mobility Assessment-Balance part; SF-12 = SF-12 health survey; FES-I = Falls Efficacy Scale-International; *p*-values from matched-pairs Student’s *t* test; ^ *p*-values from Wilcoxon matched-pairs signed-ranks test.

## Data Availability

Data supporting reported results can be found in Mendeley Data at https://data.mendeley.com/datasets/jxvhdbmmh3/1 (accessed on 2 December 2021), doi:10.17632/jxvhdbmmh3.2.

## Appendix A

Table A1 shows the value at the baseline for individual patients for the following parameters: age, gender, marital status, educational level, Hoehn and Yahr score, Rankin scale score, GDS, FAC, and MMSE.

**Table A1:** Baseline Demographic and Clinical profile.

	Age	Sex	Marital Status	Educational Level	H&Y	Rankin Scale	GDS	FAC	MMSE
**Pt 1**	80	F	Married	University	2	1	0	4	30
**Pt 2**	79	M	Married	Secondary	1	1	4	4	29
**Pt 3**	75	M	Married	Secondary	2	2	1	5	29
**Pt 4**	65	M	Married	University	2	3	4	4	24
**Pt 5**	83	M	Married	Primary	2	3	3	4	24
**Pt 6**	66	F	Married	Primary	1	1	4	5	27
**Pt 7**	77	F	Married	Primary	3	1	0	5	28
**Pt 8**	67	F	Married	Primary	2	1	1	4	26
**Pt 9**	78	M	Married	Primary	2	1	3	5	28
**Pt 10**	65	F	Married	Primary	1	1	3	5	29
**Pt 11**	68	M	Married	University	1	1	3	5	30
**Pt 12**	69	M	Married	Secondary	1	1	4	5	30
**Pt 13**	71	F	Married	Primary	1	0	3	5	26
**Pt 14**	67	F	Widowed	Secondary	1	1	0	5	26
**Pt 15**	74	F	Married	Primary	3	1	3	4	27
**Pt 16**	81	F	Widowed	Secondary	3	1	3	5	28

M = male; F = famale; H&Y = Hoehn and Yahr scale; GDS = Geriatric Depression Scale; MMSE = Mini Mental State Examination.

## Appendix B

Table A2 reports the pre- intervention values of Barthel Index, Tinetti’s Performance Oriented Mobility Assessment-Gait part, Tinetti’s Performance Oriented Mobility Assessment-Balance part, SF-12 health survey, and Falls Efficacy Scale—International for each patient.

**Table A2:** Pre-intervention score of the BI, POMA gait and Balance, SF-12, FES-I, Gait speed.

POMA			POMA			
	BI	Gait	Balance	SF-12	FES-I	Gait Speed
**Pt 1**	90	12	16	36	9	1.42
**Pt 2**	85	12	12	33	21	1.63
**Pt 3**	70	11	11	34	12	1.5
**Pt 4**	70	8	10	32	12	1.36
**Pt 5**	80	7	9	30	17	1.04
**Pt 6**	100	12	12	32	16	2.19
**Pt 7**	75	12	14	33	17	1.36
**Pt 8**	100	12	16	30	10	2.63
**Pt 9**	95	9	14	26	11	1.44
**Pt 10**	95	12	14	29	11	2.54
**Pt 11**	100	12	12	32	6	2.76
**Pt 12**	100	12	16	29	6	2.95
**Pt 13**	100	10	16	31	6	2
**Pt 14**	100	12	16	30	11	2.89
**Pt 15**	85	12	14	36	8	1.54
**Pt 16**	100	11	15	30	13	1.66

BI = Barthel Index, POMA Gait = Tinetti’s Performance Oriented Mobility Assessment-Gait part; POMA Balance = Tinetti’s Performance Oriented Mobility Assessment-Balance part, SF-12 = SF-12 health survey; FES-I = Falls Efficacy Scale–International.

## Appendix C

Table A3 reportsy, the post-intervention values of Barthel Index, Tinetti’s Performance Oriented Mobility Assessment-Gait part, Tinetti’s Performance Oriented Mobility Assessment-Balance part, SF-12 health survey, and Falls Efficacy Scale—International for each patient.

**Table A3:** Post-intervention score of the BI, POMA gait and Balance, SF-12, FES-I, Gait speed.

	BI	POMA Gait	POMA Balance	SF-12	FES-I	Gait Speed	PIADS		
							Competence Subscale	Adaptability Subscale	Self-Esteem Subscale
**Pt 1**	90	12	16	35	9	1.6	0.50	1.33	0.625
**Pt 2**	100	11	13	30	8	1.52	2.67	3.00	2.375
**Pt 3**	80	12	13	33	19	1.54	1.42	1.17	1.375
**Pt 4**	40	11	11	30	15	1.32	0.67	1.83	0.5
**Pt 5**	90	8	10	28	13	0.88	1.83	1.83	1.875
**Pt 6**	100	12	15	33	12	1.5	0.58	1.33	0.875
**Pt 7**	100	12	16	31	14	1.36	0.42	2.00	0.875
**Pt 8**	100	12	16	29	9	3.56	1.17	1.00	1.75
**Pt 9**	100	11	15	25	9	1.44	1.33	1.17	1.5
**Pt 10**	100	12	15	28	10	2.48	0.75	2.33	0.75
**Pt 11**	100	12	16	32	8	3.06	1.00	1.50	1
**Pt 12**	100	12	16	33	7	2.19	1.00	2.33	1.125
**Pt 13**	100	12	16	31	7	1.6	1.00	1.83	0.75
**Pt 14**	100	12	16	31	10	3.81	3.00	3.00	3
**Pt 15**	90	11	13	31	23	1.98	1.24	1.83	1.31
**Pt 16**	100	12	16	28	28	1.98	0.78	0.65	0.72

BI = Barthel Index, POMA Gait= Tinetti’s Performance Oriented Mobility Assessment-Gait part; POMA Balance = Tinetti’s Performance Oriented Mobility Assessment-Balance part, SF-12 = SF-12 health survey; FES-I = Falls Efficacy Scale–International; PIADS = Psychosocial Impact of Assistive Devices Scale.
